# Cytokine antibody array-based analysis of IL-37 treatment effects in asthma

**DOI:** 10.18632/aging.203515

**Published:** 2021-09-13

**Authors:** Shengnan Gao, Jingru Wang, Qing Zhang, Jun Shu, Chunxiao Li, Hongwen Li, Jiangtao Lin

**Affiliations:** 1 Graduate School of Peking Union Medical College, Chinese Academy of Medical Sciences/Peking Union Medical College, Beijing 100730, China; 2 Department of Respiratory and Critical Care Medicine, China-Japan Friendship Hospital, Beijing 10029, China; 3 Institute of Clinical Medicine Science, China-Japan Friendship Hospital, Beijing 10029, China; 4 Peking University China-Japan Friendship School of Clinical Medicine, Beijing 10029, China

**Keywords:** interleukin (IL)-37, asthma, chemokines, cytokine

## Abstract

Asthma is driven by group 2 innate lymphoid cells, antigen-specific CD4+ T helper type 2 cells and their cytokines such as interleukin (IL)-4, IL-5, IL-13. IL-37 is decreased in asthma and negatively related to Th2 cytokines and other pro-inflammatory cytokines. Our study showed that IL-37 level in asthmatic peripheral blood mononuclear cells was lower than in healthy. Further, IL-37 was negatively correlated with exhaled nitric oxide, asthma control test score, atopy and rhinitis history in asthmatics. Then an OVA-induced asthma mice model treated with rhIL-37 was established. An antibody array was employed to uncover altered cytokines induced by IL-37 in mice lung tissue. 20 proteins differentially expressed after rhIL-37 treatment and five of them were validated in asthmatic peripheral blood mononuclear cells. Consistent with cytokine antibody array, CCL3, CCL4, CCL5 decreased after IL-37 administration. While CXCL9 and CXCL13 were no change. We concluded that IL-37 reduce asthmatic symptoms by inhibit pro-inflammatory cytokine such as CCL3, CCL4, CCL5.

## INTRODUCTION

Asthma is considered to be one of the most non-communicable and prevalent chronic complications, which is characterized by variable respiratory symptoms and airflow limitation. Infiltration of various inflammatory cells, such as eosinophils, mast cells, basophils, monocytes, and lymphocytes, as well as airway hyperresponsiveness (AHR), airway remodeling, and mucus hypersecretion, are pathognomonic characteristics of asthma [1, 2]. These process are driven by group-2 innate lymphocytes, antigen-specific CD4+ T helper type 2 (Th2) cells and their cytokines including interleukin(IL)-5, IL-13, IL-4, which are capable of inducing, prolonging, and amplifying the inflammatory responses through allergic-specific immunoglobulin E (IgE) secreted by B lymphocytes [3–5].

The interleukin-1 (IL-1) family includes human IL-37 (IL-37 / IL-1F7). The coding gene of IL-37 / IL-1F7 is located on the IL-1 gene cluster 2q12-13 on human chromosome 2 [6–8]. IL-37 plays an anti-inflammation role via inhibition of the pro-inflammatory cytokines production and functions through intracellular (nuclear) and extracellular (receptor-mediated) mode. Extracellular IL-37 acts by binding to IL-18 receptor α (IL-18Rα) but does not recruit IL-18Rβ [9, 10]. In fact, IL-37 interacts with IL-1 receptor 8 (IL-1R8/ SIGIRR), the underlined event leads to the development of a tripartite complex with IL-18Rα, resulting in the initiation of anti-inflammatory signals [11–13]. Intracellular IL-37 interacts with Smad3 and transports to the nucleus, then attenuates the expression of pro-inflammatory genes at mRNA level [10, 14].

Recent findings suggest that IL-37 was decreased in serum and induced sputum of asthmatics, and its reduction was related to the severity of asthma [15–18]. In addition, the level of IL-37 was decreased in patients suffering from allergic asthma relative to nonallergic asthma patients [17]. In asthmatic mice, IL-37 treatment reduced eosinophil counts in airway tissues and BAL, reduced goblet cell hyperplasia, and improved AHR [12, 16, 19, 20]. Further, the IL-37 negatively related to Th2 cytokines in patients with asthma and allergic rhinitis, one of mechanism is through MAPK pathway [21–23]. IL-37 attenuated the development of IL-1β, IL-6, and TNF-α in sputum cells (LPS-stimulated) in asthma patients and caused suppression of IL-17 production more notably in patients with asthma than in healthy controls [15]. In epithelium and sputum-cultured cells, a partial suppression of TSLP production occurred upon the addition of recombinant IL-37 [24]. These results showed the IL-37 as a negative regulator on an allergic immune response in airways via inhibiting pro-inflammatory cytokines.

Herein, we analyze the correlation between IL-37 and clinical features in human peripheral blood mononuclear cells (PBMCs) from 19 asthma patients and 7 health. Then an advanced antibody array technology was used to evaluate protein-protein interaction network of asthmatic mice lung response to IL-37. The representative differential proteins were selectively verified in PBMCs stimulated by IL-37.

## RESULTS

### IL-37 downregulated in asthmatic PBMCs and correlated with clinical characteristics

**Figure 1:**
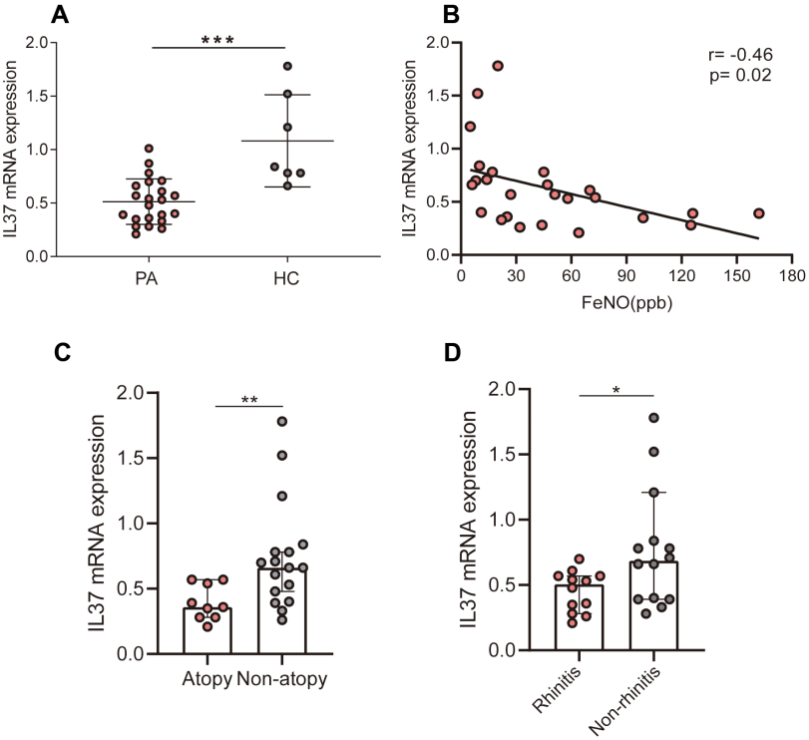
**Correlations of IL-37 levels with clinical characteristics.** Each points represents one person. (**A**) IL-37 mRNA levels of people’s PBMCs with and without asthma, as measured by means of qRT-PCR. (**B**) FeNO negatively correlated with IL-37 levels in asthma patients. (**C**) IL-37 mRNA levels of asthma patients’ (with or without allergic history) PBMCs, as measured by means of qRT-PCR. (**D**) IL-37 mRNA levels of asthma patients’ (with and without rhinitis history) PBMCs, as measured by means of qRT-PCR.

**Figure 2:**
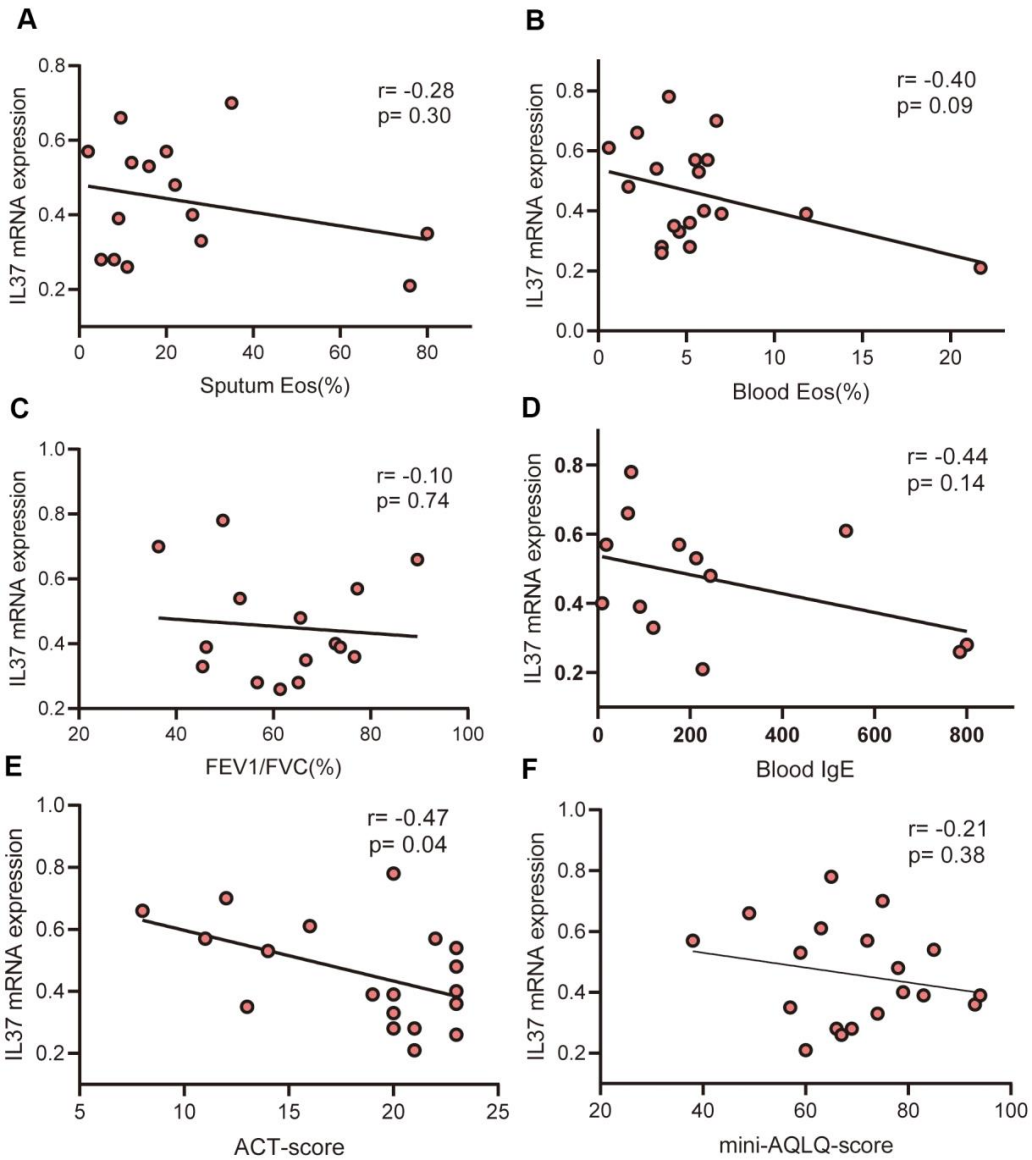
**Correlations of IL-37 levels with clinical characteristics.** Each points represents one person. (**A**) There is no correlation between IL-37 mRNA levels and % sputum eosinophils (r=-0.28, p=0.30). (**B**) There is no correlation between IL-37 mRNA levels and % peripheral blood eosinophils (r=-0.40, p=0.09). (**C**) There is no correlation between IL-37 mRNA levels and %FEV1/FVC (r=-0.10, p=0.74). (**D**) There is no correlation between IL-37 mRNA levels and peripheral blood IgE (r=-0.44, p=0.14). (**E**) ACT-scores were negatively correlated with IL-37 mRNA levels (r=-0.74, p=0.04). (**F**) There is no correlation between IL-37 mRNA levels and mini-AQLQ-scores (r=-0.28, p=0.30). Data were analyzed using Spearman’s rank correlation.

In the current study, IL-37 mRNA was identified in PBMCs from 19 patients with asthma (PA) and 7 healthy control (HC), whose clinical characteristics have shown on Table 1. We demonstrated marked downregulation of IL-37 in PA relative to that in HC (Figure 1A). Furthermore, the link between the level of IL-37 and clinical features was evaluated by Spearman correlation analysis. There was a negative correlation between IL-37 level and FeNO (r=-0.46, P=0.02) (Figure 1B). IL-37 expression in asthma patients with atopy history (Atopy) was lower than that without atopy history (Non-atopy) (Figure 1C). Similarly, IL-37 expression of asthma patients with rhinitis (Rhinitis) was lower than that without rhinitis (Non-rhinitis) (Figure 1D). However, there was no correlation between IL-37 and percentage of sputum eosinophils (r=-0.28, p=0.30) (Figure 2A), percentage of blood eosinophils (r=-0.40, p=0.09) (Figure 2B), FEVI/FVC (r=-0.10, p=0.74) (Figure 2C), blood total IgE (r=-0.44, p=0.14) (Figure 2D). In terms of clinical symptoms, IL-37 expression of asthma patients was negatively correlated with ACT-score (r=-0.47, p=0.04) (Figure 2E) but no correlation with mini-AQLQ-score (r=-0.21, p=0.38) (Figure 2F).

**Table 1:** Participants’ characteristic.

	**HC, n=7**	**PA, n=19**	**P value**
Age(years), mean(SD)	37.57(16.47)	37.20(14.77)	0.737
Sex, female n(%)	4(57)	10(53)	0.5
BMI, mean(SD)	23.37(4.36)	24.53(6.20)	0.304
FEV1/FVC, mean(SD)	-	64.67(4.06)	-
FeNO(ppb), geo mean(SD)	11.57(5.62)	56.3(6.33)	0.000
Sputum eos.%, median(IQR)	0(0.00,0.00)	16.00(9.00,28.00)	0.000
Secrum eos.%, median(IQR)	-	5.20(3.60,6.20)	-
ACT-score, median(IQR)	-	20.00(14.00,23.00)	-
Mini AQLQ-score, mean(SD)	-	65.00(13.81)	-
Secrum total IgE, (KU/L), median(IQR)	-	176.00(68.75,391.00)	-
Methacholine, n positive(%)	0(0)	4(21.05)	0.001
Atopy, n(%)	0(0)	9(47.37)	0.030
Rhinitis, n(%)	0(0)	12(63.16)	0.030

BMI, Body Mass Index; FEV1, Forced expiatory volume in 1st second; FVC, Forced vital capacity; FeNO, Fractional exhaled nitric oxide; ACT, asthma control test; Mini-AOLQ, mini asthma quality of life questionnaire; HC, Healthy Control; PA, Patients with Asthma.

### IL-37 attenuated asthmatic symptoms and eosinophils infiltration in mice model

**Figure 3:**
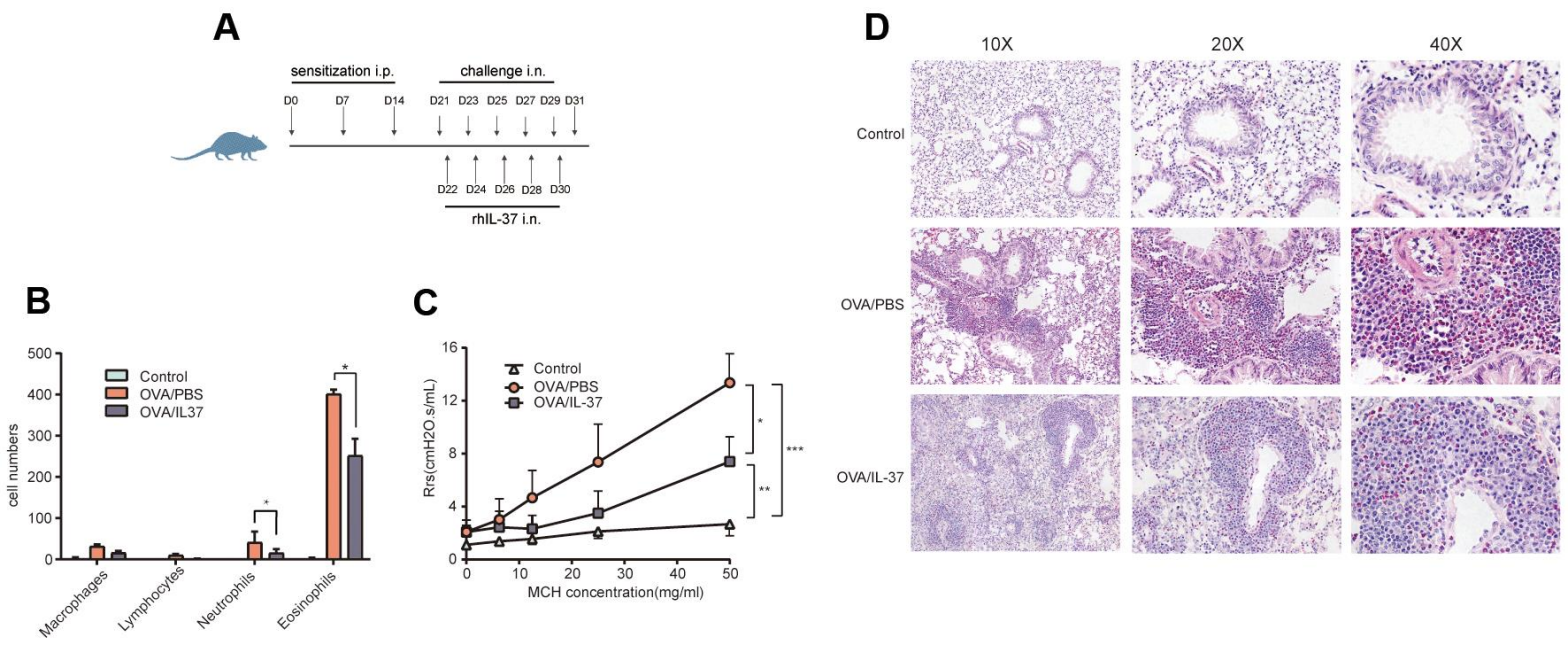
**IL-37 expression and the administration of rhIL-37 in the challenge phase attenuates OVA-induced eosinophilic airway inflammation and airway hyper-reactivity (AHR).** (**A**) Protocol for OVA-induced asthmatic airway inflammation and the time points of rhIL-37 intervention during the sensitization phase. (**B**) The differential cell counts in BALF (n=10 mice per group). Numbers of cells were counted in ten random 1000X oil lens fields. (**C**) Airway resistance to methacholine was measured at 24 hours after final OVA challenge by using Flexivent FX-Mouse AN modular and invasion system (n=10 mice per group). (**D**) Representative photomicrographs of lung sections stained with H&E. original magnification, 10x, 20x, 40x. Columns and error bars represented mean±SEM. n=10 per group. *p< 0.05, **p< 0.01, ***p<0.01. Similar results were obtained from three to five independent experiments.

A contribution of rhIL-37 in asthma mouse models (caused by OVA) was confirmed in this section. The modeling process is shown in Figure 3A. OVA considerably elevated AHR compared to Control, which was lowered by rhIL-37 (Figure 3B). In addition, the number of lymphocytes, macrophages, eosinophils, and neutrophils in OVA/PBS animals BALF was higher than in Control mice. rhIL-37 reduced these cell counts especially eosinophils (Figure 3C). In addition, H&E staining showed more neutrophils and eosinophils infiltration in the OVA/PBS relative to Control, and IL-37 treatment reduced the eosinophils infiltration compared with OVA/PBS (Figure 3D).

### IL-37 altered receptor expression in mice lung tissue

**Figure 4:**
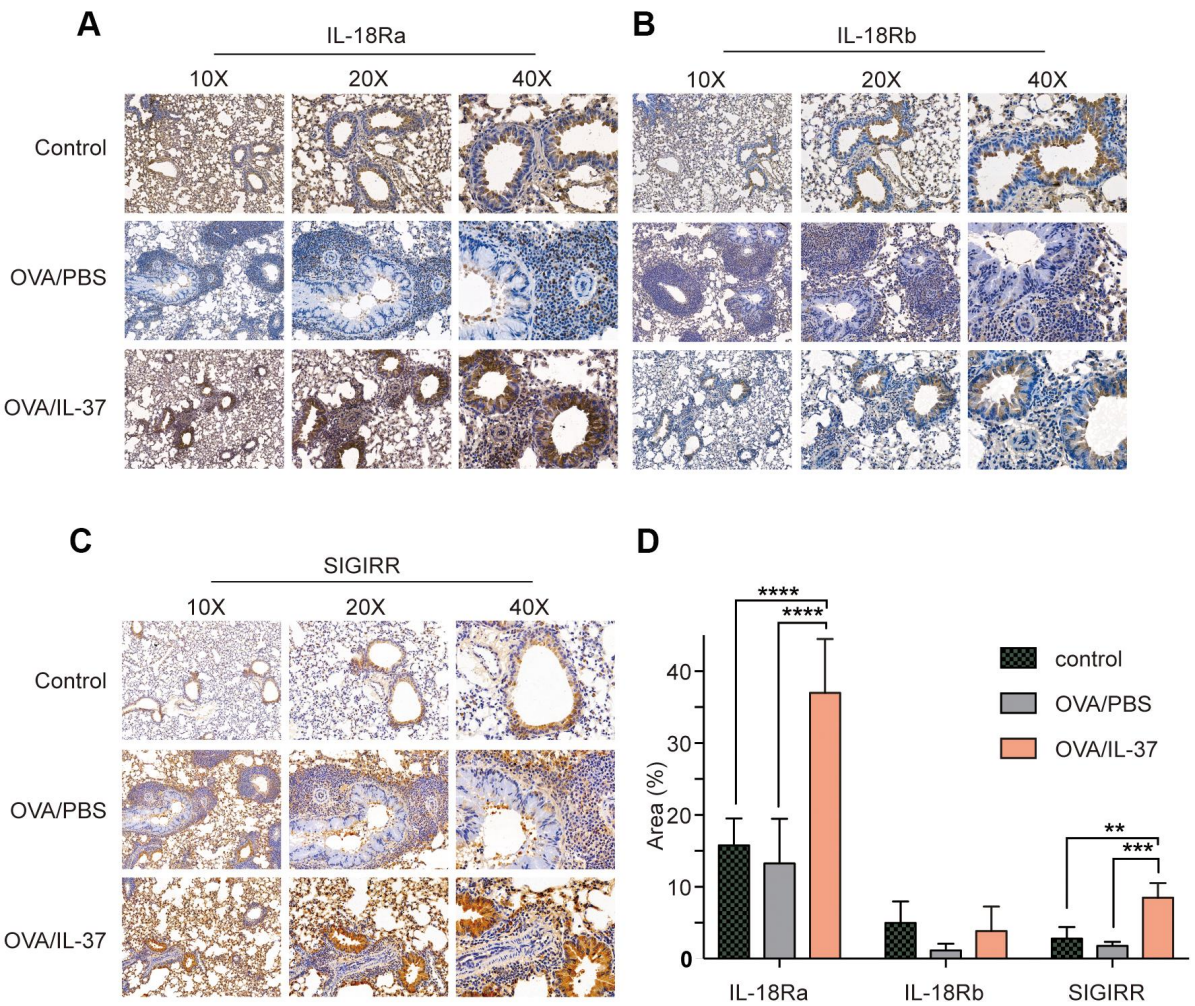
**Immunohistochemistry detection of IL-18Ra, IL-18Rb, SIGIRR in mice lung tissue from PBS, OVA/PBS, OVA/rhIL-37 group (n=10 mice per group).** Representative photomicrographs of immunohistochemistry staining for IL-18Ra (**A**), IL-18Rb (**B**) and SIGIRR (**C**) in mice lung tissue from each group. (**D**) Quantitation of IL-18Ra, IL-18Rb, SIGIRR expression. IL-18Ra and SIGIRR expression in the lung tissue of the OVA/IL-37 group was significantly upregulated compared to the OVA/PBS group, while the difference of IL-18Rb expression in the lung tissue of the OVA/IL-37 group was not statistically significant upregulated compared to the OVA/PBS group. Columns and error bars represented mean±SEM. n=10 per group. **p< 0.01, ***p<0.001. ****p<0.0001.

IL-18Ra is the key receptor of IL-37 function. Unlike the way IL-18 works, IL-37 recruits SIGIRR after binding to IL-18Ra instead of IL-18b. Immunohistochemistry of these receptor of mice lung showed that IL-18Ra expression was no difference after OVA challenge, but increased after IL-37 treatment compared with OVA/PBS (p<0.0001) and Control (p<0.0001) (Figure 4A, 4D). The same trend and subcellular localization with IL-18Ra could be seen in SIGIRR expression (p<0.01) (Figure 4C, 4D). On the contrary, IL-18b level was no difference between each group(p>0.05) (Figure 4B, 4D).

### Altered cytokine levels after IL-37 treatment

**Figure 5:**
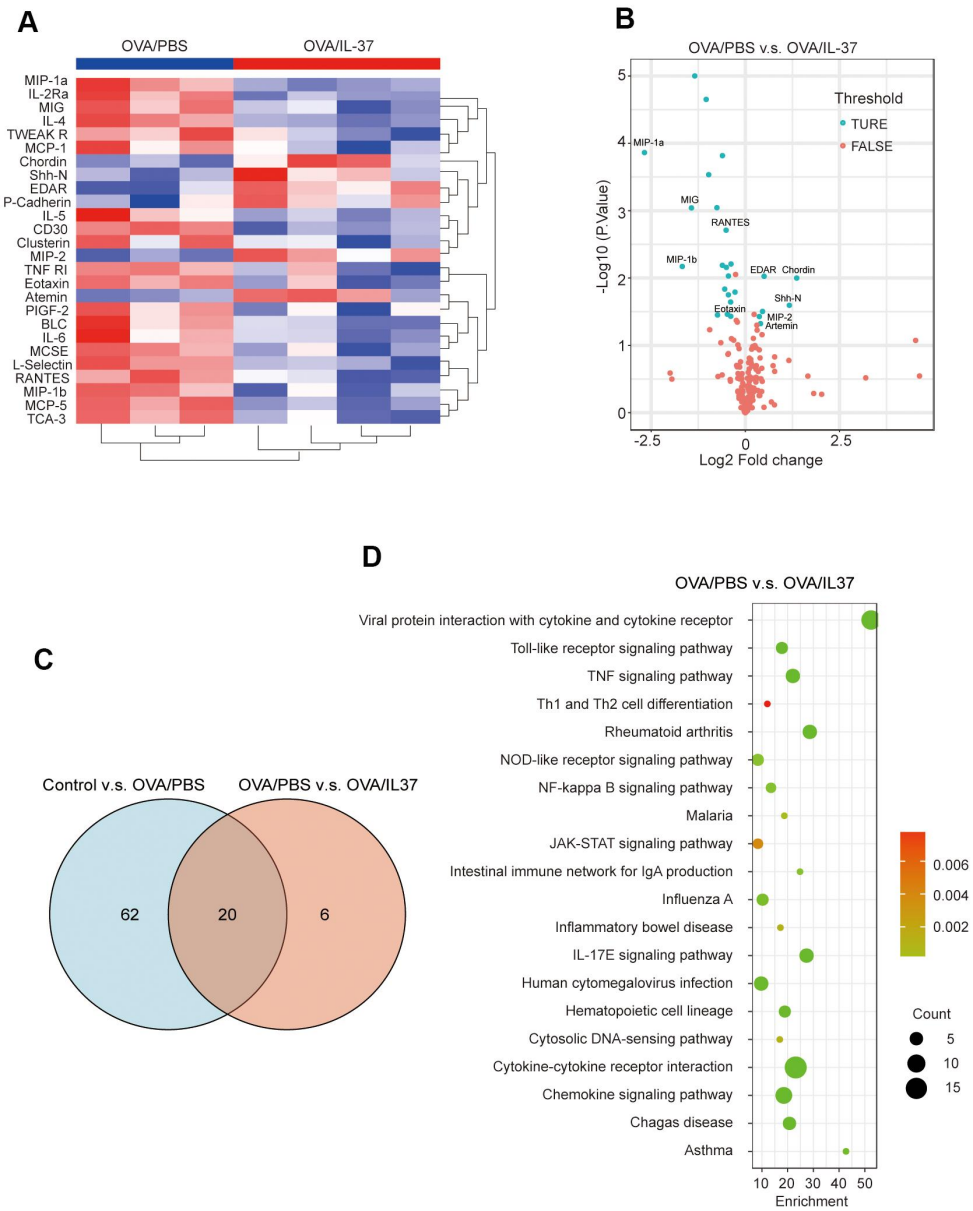
**Visualization of cytokine antibody array analysis.** (**A**) Clustering heatmap. Red represents OVA/IL-37 group, blue represents OVA/PBS group. (**B**) Volcano plot shows 26 differentially expressed proteins (DEPs)(blue dot) between OVA/PBS and OVA/IL-37, which are defined as those with adjust p value(adj.P.Val) less than 0.05 and foldchange over 1.2 or less than 0.83(absolute LogFC>0.263). Top 10 DEPs have been marked on the picture. (**C**) There are 20 DEPs result from intersection of Control v.s. OVA/PBS and OVA/PBS v.s. OVA/IL-37. (**D**) Protein function annotation KEGG pathway (OVA/PBS v.s. OVA/IL-37).

The basic statistics used for significance analysis were moderated t-statistic. Differentially expressed proteins (DEPs) were defined as those with adjusted P-value less than 0.05, and fold change over 1.2 or less than 0.83, which were presented as Volcano plot (Figure 5B and Supplementary Figure 1A). All of the DEPs were analyzed by cluster analysis between Control and OVA/PBS (Supplementary Figure 1B), or OVA/PBS and OVA/IL-37 (Figure 5A). The intersection between two groups represents these molecules up/down-regulated in asthmatic mice but down/up-regulated after rhIL-37 administration (Figure 5C), MIP-1a(CCL3), MIP-1b(CCL4), MIG(CXCL9), RANTES(CCL5), BLC(CXCL13) etc. in 20 of them. In addition, protein function annotation Gene Ontology (GO) and KEGG pathway were evaluated (Figure 5D and Supplementary Figure 1C–1E).

### Elisa validation results

**Figure 6:**
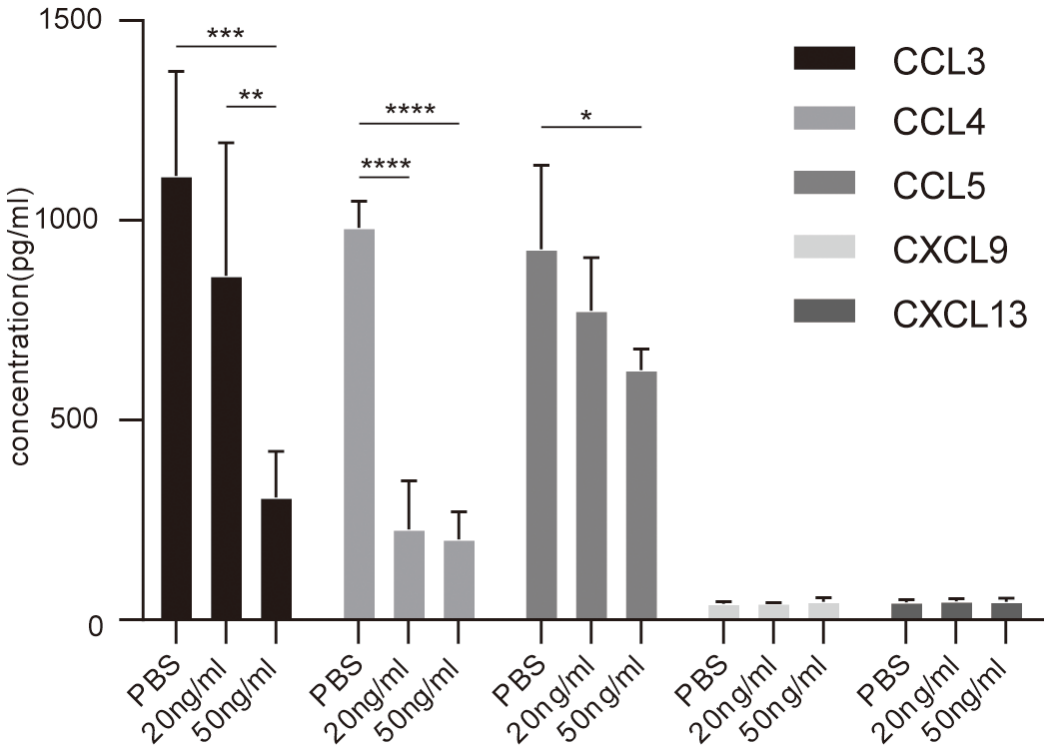
**Elisa analysis of chemokines concentration in PBMCs culture supernatant with or without IL-37 treatment.** Columns and error bars represented mean±SEM. n=6 per group. *p< 0.05, **p< 0.01, ***p<0.001. Similar results were obtained from three independent experiments.

In order to validate the differential expression proteins affected by IL-37, Elisa was performed MIP-1a(CCL3), MIP-1b(CCL4), RANTES(CCL5), MIG(CXCL9), BLC(CXCL13) in asthmatic PBMCs stimulated by rhIL-37 (Table 2). The results showed that MIP-1a, MIP-1b, RANTES down-regulated after 24 hours rhIL-37 treatment. MIG and BLC presented a low level and had no significant change after rhIL-37 administration (Figure 6).

**Table 2:** PBMCs donors’ information.

**Name**	**Sex**	**Age** **(years)**	**BMI** **(kg/cm^2^)**	**FEV1/FVC** **(%)**	**Blood eos(%)**	**Sputum eos(%)**	**Secrum IgE(KU/L)**	**FeNO (ppb)**
P.LI	male	33	29.90	58.17	5.2	30	99	44
PF.Mao	male	38	37.70	77.25	4.6	28	120	22
GL.Liu	female	43	22.30	49.61	6.2	20	176	51
SM.ZH	female	32	23.40	75.78	5.5	19	176	47
HY.Ton	female	28	20.40	79.40	4.8	37	143	32
QQ.Nie	male	38	29.4	53.12	4.3	40	111	54

BMI, Body Mass Index; FEV1, Forced expiatory volume in 1st second; FVC, Forced vital capacity; FeNO, Fractional exhaled nitric oxide.

## DISCUSSION

Reported studies have been revealed that the level of IL-37 has been lowered in asthma patients relative to the healthy controls [15–18]. The level of IL-37 was lowered in patients suffering from allergic asthma relative to nonallergic asthma [17]. In our study, we detect IL-37 mRNA of PBMCs from 19 asthma patients and 7 healthy volunteers. The level in asthma patients was lower than that in healthy control which consistent with previous studies. This implies that the loss of IL-37 function may be a part of the pathogenesis of asthma and in the majority of inflammatory diseases. Further, we evaluated the correlation between the expression of IL-37 and clinical features in human PBMCs from 19 asthma patients and 7 health, which has not been clarified in previous studies. The obtained results revealed that IL-37 expression was negatively correlated with FeNO, ACT-score, atopy and rhinitis history.

Previous studies told us that extracellular IL-37 interacts with IL-18Rα on the cell surface. Different from the way IL-18 works, IL-18Rα does not recruit IL-18R β, but IL-1R8 to transfer anti-inflammatory signals [11, 12]. Immunohistochemistry of these receptor of mice lung showed that IL-18Ra and SIGIRR expression in OVA/PBS group decreased compared with control mice, but increased after IL-37 treatment, while IL-18b expression in OVA/IL-37 group was no difference with OVA/PBS group. These were consistent with previous results in the aspect of extracellular receptor binding function.

Asthma is an inflammatory disease of the lower airway caused by a mix of environmental factors, genetic predisposition, and perhaps metabolite and microbiome changes [25]. Type 2 inflammation (type 2 T helper cell lymphocyte) is common in asthma patients, and it is linked to certain inflammatory cells (mast cells, eosinophils, type 2 T helper cell lymphocyte, IgE-producing plasma cells, and basophils) and cytokine profiles (IL-13, IL-5, IL-4) [26]. In the present study, we utilized traditional methods such as AHR measurement and histological examination to authenticate an asthma model. Additionally, cytokine antibody array revealed that IL-37 could result in reduced expressions of MCP-5, IL-4, IL-5, IL-2Ra, MIP-1a(CCL3), MIP-1b(CCL4), TCA-3(CCL1), MCSF, MIG(CXCL9), RANTES(CCL5), BLC(CXCL13), IL-6, L-Selectin, MCP-1, CD30, TNF RI, Clusterin, Eotaxin, PIGF-2, and elevated expressions of EDAR, Shh-N. As reported previously, among those biomarkers, monoclonal antibodies against IL-4 and IL-5 have been developed for the treatment of asthma [27, 28]. IL-6 [29, 30], RANTES [31], Clusterin [32–35], MIG [36], BLC [37] and MIP-1a/b [38] have been found to exhibited raised levels in asthma. However, their relationship with IL-37 has not been reported. Our bioinformatics analysis of these factors showed the enriched KEGG pathways terms included “TNF signaling cascade”, “NOD-like receptor signaling cascade”, “NF-kappa B signaling cascade”, “JAK-STAT signaling cascade”, and “IL-17E signaling cascade”. The underlined cascades have a role in the immune response of asthma, and IL-37 may regulate the progression of asthma through these proteins and pathways.

At last, we validate the expression of MIP-1a(CCL3), MIP-1b(CCL4), RANTES(CCL5), MIG(CXCL9), BLC(CXCL13) in human PBMCs from asthmatics after rhIL-37 treatment. We observed that after IL-37 stimulation for 24 hours, the elevated levels of MIP-1a(CCL3), MIP-1b(CCL4), RANTES(CCL5) decreased, which consistent with previous studies and cytokine antibody array. MIG(CXCL9) and BLC(CXCL13) with lower levels did not change. A meta-analysis published in 2020 revealed that RANTES(CCL5) -403G/A and -28C/G genetic polymorphisms significantly contribute to the development of childhood asthma [31]. Several studies have described the CCL5-dependent recruitment of eosinophils during allergic airway inflammation. Those effects were decreased by neutralization of CCL5 receptors [39]. In our study, CCL5 secretion from 6 asthmatic PBMCs was inhibited by IL-37, which also consistent with previous studies and the performance of cytokine antibody array.

The expression of CXCL9 and CXCL13 showed a low level in PBMCs, and there was no significant change after IL-37 treatment. The underlying mechanism of allergic asthma is the evidence that appear to be large and localized in nature, but that these events may appear at systemic levels [40]. The reason may be related to the difference between systemic (PBMCs) and local (mice lung tissue) effects.

However, there are several limitations in our study. The number of enrolled patients is relatively small, and its relationship with clinical characteristics need fully clarified. The correlation between them requires a larger sample size to confirm. In addition, we should note that chemokines are not only expressed functioned in leukocytes, but also in structural cells. The expression of these cytokine might need to be further verified and explored in airway epithelial cells, smooth muscle cells and (myo)fibroblasts.

## Conclusion

In asthma patients, the expression level of IL-37 was decreased in PBMCs relative to healthy people. IL-37 was also found to be negatively correlated with FeNO, ACT-score, atopy and rhinitis history in asthmatic adults. Bioinformatic analysis revealed 20 proteins differential expression after rhIL-37 treatment. MIP-1a(CCL3), MIP-1b(CCL4), RANTES(CCL5) from asthmatic PBMCs decreased after rhIL-37 stimulation, while CXCL9 and CXCL13 production remains unchanged.

## MATERIALS AND METHODS

### Animals and ethical approval

Experiments were approved by the China-Japan Friendship Hospital Animal Experimental Ethics Committee in Beijing, China. 6-8 weeks female BALB/c were procured from Vital River Laboratory Animal Technology Co. Ltd (China) and were housed in a pathogen-free environment in clinic research institute of China-Japan Friendship Hospital, Beijing, China. Mice were kept in a 12-hour light-dark cycle with ad libitum access to food and water.

Thirty mice were randomly categorized into three groups i.e., the Control, OVA/PBS, and OVA/IL-37 groups. In OVA/PBS group, the mice were sensitized by intraperitoneal injection of ovalbumin (OVA, Sigma-Aldrich, Beijing, 100ug emulsified in Al (OH)_3_ /dose) on day 0, day7, day14, then further challenged every other day per-nasally from day 22 to day 30 with 100ug of OVA in 50uL PBS/dose. In the Control group, an equal amount of Al (OH)_3_ (intraperitoneally) was given to mice and then nasally challenged with PBS at the same time points as the actively challenged mice (Figure 1A). The mice in OVA/IL-37 group were treated with rhIL-37 (R&D System, USA, 200ng/dose) or with PBS as control 24h before OVA administration.

### Measurement of asthma mice model

Whole-body plethysmography (SCIREQ, Canada) was used for the determination of AHR after 24 h of the final intranasal OVA challenged. The calculation of the mean Rrs was carried out from measurements during a 5 min period after methacholine chloride (MedChemExpress, USA) (3.125-50mg/ml) inhalation. Mice were euthanized with pentobarbital (1.5%) after AHR measurement, and BALF was taken to determine differential BALF cell count. The right lung lobes were washed thrice with 1.4 ml ice-cold PBS as BALF. The samples of BALF were then subjected to centrifugation for 5 min at 400g for the collection of cells pellet. This was followed by the supernatant collection and storage at -80° C until use. Further, re-suspension of the cell pellets was carried out with PBS to determine differential cell counts through the Wright’s-Giemsa staining. The fixation of the left lung was carried out with paraformaldehyde (4%) overnight at 4° C followed by its embedding in paraffin. Staining of paraffin sections (5 μm) was performed with hematoxylin and eosin (H&E) for eosinophilic infiltration detection.

### Immunohistochemistry

Lung sections were dehydrated in graded alcohol solutions after being treated with xylene. H_2_O_2_ (3%) was used to inhibit endogenous peroxidase activity. To minimize nonspecific immunoglobulin absorption, tissues were flooded with normal goat serum (5%), then incubated for 90 minutes with anti-IL-18Rα, anti-IL-18Rβ, anti-SIGIRR polyclonal antibody (Abcam, USA) at a dilution of 1:5000As a negative control, PBS was substituted for each primary antibody. Overnight incubation of slides was carried out at 4° C, followed by three times rinsing with PBS. Furthermore, the incubation of the underlined sections was carried out with secondary antibodies labeled with peroxidase (DAKO, Glostrup, Denmark) at room temperature for 0.5 hours, followed by washing with PBS before being stained with diaminobenzidine. The sections were then dehydrated and viewed after being counterstained with hematoxylin. Data are represented as the percentage of positive staining area in total.

### Cytokine antibody array

A mouse cytokine antibody array (Mouse Cytokine Array GS4000, Raybiotech lnc, USA) was employed to simultaneously detect and quantify 200 cytokines in lung sample collected from asthma mouse model and rhIL-37 treated asthma mouse model. 1ml 1X Cell Lysis Buffer (with Protease Inhibitor Cocktail) was used to homogenize the tissue sections. After 30 minutes’ tissue lysis, the centrifugation (at 13,000 rpm) of samples was carried out for 20min, followed by collecting the supernatant, and then the protein levels were evaluated. The visualization of the signals can be performed using a laser scanner (InnoScan 300 Microarray Scanner, France) that has a Cy3 wavelength (green channel). Lung samples from 5 mice per experimental group were analyzed.

### Human samples collection

Human peripheral blood was collected from patients with asthma (PA) (n=19) and healthy control (HC)(n=7) with matched age and sex recruited from pulmonary outpatient department of China-Japan Friendship Hospital in Beijing. The diagnosis of asthma was made in accordance with the Global Initiative for Asthma guidelines, all subjects with symptoms of respiratory tract infection in previous 3 weeks, and with peroral steroid treatment in previous 3 months, and with smoking history were excluded. Informed consent was provided by each participant included in this study. Serum samples were obtained by centrifugation of 12ml venous blood. The serum separation was achieved through 15 minutes’ centrifugation at 1,000×g followed by its storage at -80° C. Ficoll density gradient (Amersham Biosciences) was used for the isolation of PBMCs from samples of blood collected in EDTA-coated vacutainer tubes (BD, Biosciences, Canada).

### Clinical information and examination of patients

Pulmonary function tests, FeNO (Fractional Exhaled Nitric Oxide), Percentage of eosinophils in induced sputum, Methacholine airway provocation test were obtained from Clinical Diagnosis Department of Respiratory Diseases Center, China-Japan Friendship Hospital, Beijing, China. Percentage of eosinophils in secrum, Secrum total IgE were detected by Laboratory Department of China-Japan Friendship Hospital in Beijing, China.

### Quantification of IL-37 mRNA using real-time PCR

After PBMCs isolation, TRIzol Reagent (Invitrogen, USA) was used to extract the total RNA. All step were performed according to manufactures’ instructions. 1μg of total RNA was reversely transcribed into cDNA via ReverTra Ace RT Master Mix with gDNA Remover (TOYOBO, Japan). RT-PCR was carried out on the ABI 7500 RT-PCR system using SYBR Green (DBI Bioscience, Germany). The primers employed in the current study are given below: IL37 forward:5’- TTCTTTGCATTAGCCTCATCCTT-3’, reverse: 5’-CGTGCTGATTCCTTTTGGGC-3’ GAPDH forward: 5’-CCGGTACTCGTTTGACTCCT-3’, reverse:5’-TGCTTCACCACCTTCTTGATG-3’. The calculation of the target gene relative expression was carried out using the 2^-ΔΔ CT^ method. Normalization was achieved with GAPDH.

### Cytokine concentration measurements

ELISA kits were used for the determination of CCL3, CCL4, CCL5, CXCL9, CXCL13 in human serum according to manufacturer’s instructions (Multisciences, China).

### Statistical analysis

All data were analyzed using non-parametric tests using the software GraphPadPrism, and all data were expressed as mean SEM (version 7.0a). When comparing more than two groups, a one-way ANOVA with multiple comparison tests was employed. To compare variation between groups, the two-tailed Mann-Whitney test was employed. Spearman correlation analysis was applied to determine the correlation between IL-37 and clinical features. P-values < 0.05 were regarded as statistically considerable.

## Supplementary Material

Supplementary Figure 1
