# Supplementary material for: Cytokine antibody array-based analysis of IL-37 treatment effects in asthma
Source: Aging (Albany NY). 2021 Sep 13;13(17):21729–42. doi: 10.18632/aging.203515 (PMC8457575; doi:10.18632/aging.203515)
Supplement: Supplementary Figure 1 [file aging-13-203515-s001.pdf]

SUPPLEMENTARY FIGURE

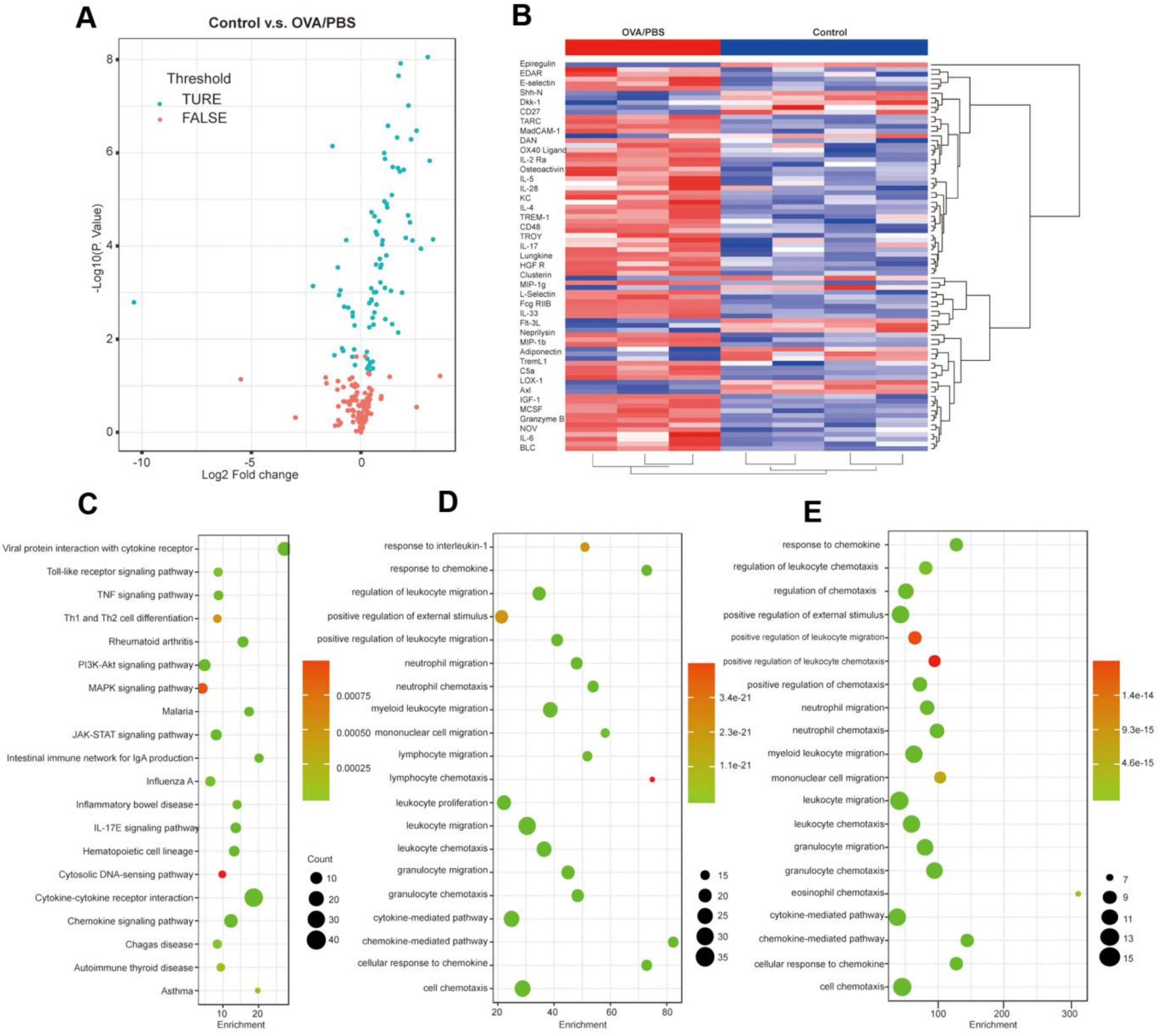

Supplementary Figure 1. (**A**) Volcano plot shows 82 differentially expressed proteins (DEPs) (blue dot) between Control and OVA/PBS, which are defined as those with adjust p value(adj.P.Val) less than 0.05 and foldchange over 1.2 or less than 0.83(absolute LogFC>0.263). (**B**) Clustering heatmap. Red represents OVA/PBS group, blue represents Control group. (**C**) Protein function annotation KEGG pathway (Control v.s. OVA/PBS). (**D**) Protein function annotation Gene Ontology (GO)-BP (biological process) subtype (Control v.s. OVA/PBS). (**E**) Protein function annotation Gene Ontology (GO)-BP (biological process) subtype (OVA/PBS v.s. OVA/IL-37).
